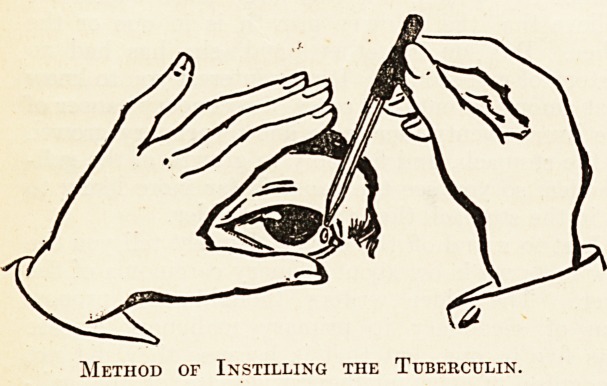# The Ophthalmo-Tuberculin Reaction in Private Practice

**Published:** 1908-04-25

**Authors:** 


### SPECIAL ARTICLE.

### THE OPHTHALMO-TUBERCULIN REACTION IN PRIVATE PRACTICE.

The Wolf-Eisner-Calmette ophthalmo-tuberculin reaction has now been in use for more than three years, and its application is so easy, while the results obtained are often so helpful, that it is well worth the attention of the general practitioner. As a method of diagnosis it is, of course, not ideal. Only the student, in his first few months of ward work, dreams of a short cut towards diagnosis—the penny-in-the-slot method of making sure. Afterwards, when he comes to realise that diagnosis, to be exact, is not a matter of one sign or two, but the careful correllation and consideration of many subjective and objective details and that it involves a close attention to minor points, he learns that such ready-made methods are as fallacious as they are undesirable. Nevertheless, while bearing this in mind, the practitioner should consider sympathetically any new means which is brought to his notice as a possible aid towards simplifying diagnosis. The knowledge of the use of a simple test is often of service in elucidating a doubtful case, and even where

Method of Instilling the Tuberculin.

its result is purely a negative one some useful information will have been gained.

Both Calmette and Wolf-Eisner found that when tuberculin was dropped upon the sensitive mucous membrane of a tuberculous patient there ensued in the course of a short interval a definite hyperæmia and transient inflammation of the mucosa at the site of application. Von Pirquet, at an earlier date, had found that tuberculin rubbed in on a sensitive part of the skin produced a similar inflammatory reaction unattended with the severe constitutional disturbance that follows a subcutaneous injection of tuberculin. Since the publication of the original papers by Calmette and Wolf-Eisner the method has been closely investigated by many observers, both on the Continent and abroad, and the general results have amply confirmed Calmette's original assertion that the reaction is fairly constant, and verified his hope that it would prove a diagnostic method of value in diagnosing cases of latent tubercle. Stephenson and later Eyre, in England, have published the results of their findings in a series of cases, and as modified at present the method is so simple that it can be followed by any practitioner. In many cases it has been used in private practice with excellent results.

The method consists essentially in dropping into the conjunctival sac of one of the patient's eyes an attenuated tuberculin. This tuberculin is put up either in the form of discs or as a solution in tiny glass tubes which may now be obtained from most large firms of manufacturing chemists. The Clinical Research Association puts up test solutions, with full instructions; and the original Calmette dilution may be obtained from the Pasteur Institute at Lille, or from Messrs. Ponleuc Frères, of Paris. It is usually supplied in a sealed glass tube, furnished with a small rubber squeezer. The tip of the tube is broken off and the solution gently dropped on to the everted lower eyelid, as shown in the diagram.

The eyelid is closed for a short time, and a note is made of the time and site of injection, whether right or left eye. If a positive result (denoted as O.R. +) follows, the bulbar conjunctival reflexion is found to be slightly reddened, usually after a lapse of from 18 to 24 hours. A few hours later the caruncle becomes definitely hyperæmic as compared with that of the uninjected eye, and in the course of 48 hours a more or less well-marked conjunctivitis results. This is usually transient and disappears as rapidly as it has originated. The patient suffers little inconvenience, generally nothing more than a slight lachrymation. In case of a negative result no difference is to be noticed in the injected eye after 48 hours. In such cases a second instillation may be tried.

With proper care, the method is free from danger. In a few cases the reaction has been excessive, leading to an acute conjunctivitis which has had to be treated; but such results are very rare, especially with the tuberculins now supplied. The objections to the method are that it is so often successful in cases which have no obvious tuberculous lesion, and that it fails in cases of advanced tuberculosis where the patient is almost moribund, as in acute general tuberculosis. For practical purposes, however, its use lies more in its value as a confirmative test than in its value as a special test. Cases of doubtful tubercular abscesses, swellings which are not exactly demonstrably tubercular, and early lung cases are those in which the practitioner will find it of use.

Sometimes the positive reaction is exceedingly slight and transient, and may therefore be overlooked by the physician. The eye should be systematically examined at intervals after the instillation, particular attention being paid to the caruncle and to the bulbar reflection of the conjunctiva at its inner margin, and should be compared with the uninjected eye.

In Germany a modification of this reaction is now employed, a salve being used containing a mixture of tuberculin, lanolin, and olive oil. Calmette has used a similar test for typhoid fever, employing an attenuated typhoid serum as the material for dropping into the eye, and has reported favourably on it.

## Figures and Tables

**Figure f1:**